# Differences in Clinical Outcomes and Patients' Characteristics Between Childhood and Adolescent Testicular Torsion

**DOI:** 10.7759/cureus.75245

**Published:** 2024-12-06

**Authors:** Takahiro Hosokawa

**Affiliations:** 1 Radiology, Saitama Children's Medical Center, Saitama, JPN

**Keywords:** acute scrotum, children, emergency, pediatric, testicular torsion

## Abstract

Objectives: Testicular torsion, a condition requiring urgent intervention, can occur at any age and present with diverse symptoms. To the best of our knowledge, no study has evaluated the characteristics of testicular torsion in childhood, a less common age group. This study showed differences in patients’ characteristics between childhood and adolescence and the variation across ages.

Methods: The 58 patients included were classified into two groups: childhood (1-9 years) and adolescence (10-16 years). The following patient characteristics were compared between the two groups using Fisher’s exact and Mann-Whitney U tests: presence or absence of acute scrotum as the first symptom, period from symptom onset to diagnosis, and whether testicular salvage was achieved. Single linear regression analysis was used for testicular salvage and acute scrotum as first symptom rates to evaluate the relationship between these factors and each age every one year.

Results: Testicular torsion was observed in childhood and adolescence in 11 and 47 of the patients, respectively. The average period from first symptom onset to diagnosis was 19.97±24.31 (range, 2-72) hours, with acute scrotum as the first symptom observed in 43 patients and testicular salvage in 39. Significant differences were observed between the childhood and adolescence groups regarding the period from first symptom onset to diagnosis, presence/absence of acute scrotum as the first symptom, and testicular salvage/not salvage. Additionally, strong correlations between patient age and both testicular salvage and acute scrotum as first symptom rates were observed.

Conclusions: Testicular torsion in childhood tends to present without typical symptoms as the acute scrotum, leading to delayed diagnosis and lower testicular salvage rates of the affected testis.

## Introduction

Acute scrotum is a common presenting symptom in emergency departments [1,2]. In this situation, testicular torsion is an important condition in pediatric patients requiring urgent intervention [3,4]. Additionally, patients with testicular torsion may present with various initial symptoms, such as abdominal pain or vomiting, rather than acute scrotum [1-6]. Among these atypical symptoms, abdominal pain was the most common symptom (6%), while nausea and vomiting were the least symptoms [7]. Diagnosis delays lead to testicular ischemia, which can result in testicular loss [3]. Testicular loss can usually be avoided if the detorsion is performed within six hours [8]. Therefore, rapid diagnosis is critical to prevent this complication.

Patient age and clinical symptoms are essential in diagnosing testicular torsion [4,9]. Testicular torsion incidence has two peaks: infancy (0-1 year) and adolescence (10-19 years); in Japan, the largest incidence is observed among those aged 10-16 years [3,5]. The incidence rate of testicular torsion was 6/100000 person-years in boys aged 5-9 years and 30/100000 person-years in those aged 10-14 years [4].

Previous studies have focused on differences in patient characteristics of testicular torsion in infancy (perinatal testicular torsion) and adolescence, as well as in pre- and post-pubertal males [3-6,10-12]. However, testicular torsion can occur at any age, including childhood [3-5]. To the best of our knowledge, no study has evaluated the characteristics of testicular torsion in childhood (1-9 years), a less common age group, or how these differ by age. Therefore, this study aimed to show differences in patient characteristics between patients in childhood (1-9 years) and in adolescence (10-16 years) as well as age-based differences.

## Materials and methods

Ethical considerations

This retrospective study was conducted in accordance with the tenets of the Declaration of Helsinki and approved by the Saitama Children’s Medical Center ethics committee (approval number 2023-03-005). The requirement for informed consent was waived due to the study’s retrospective nature.

Study population

We searched electronic medical records for patients treated at our institution (7000 pediatric patients/per year attending the pediatric emergency department) over 15 years, between May 2007 and August 2023, identifying 71 children aged 1-16 years with clinically and surgically diagnosed testicular torsion.

The exclusion criteria were as follows: patients with undescended testicular torsion (n = 3), patients with undescended testicular torsion clinical course that differed from that of typical testicular torsion [13], and those with unavailable medical records (n = 10).

Patient classification

In Japan, the highest incidence was observed among those aged 10-16 years, whose ages were close to puberty [4,7]. Additionally, patients aged <10 years were usually in the prepubertal stage. Testicular torsion usually occurs in patients during puberty [5,9]. Therefore, in our study, patients were classified into the following two groups: childhood (1-9 years) and adolescence (10-16 years).

Patients’ characteristics

All pediatric patients were diagnosed based on physical, sonographic, and surgical findings [14,15]. The following patients’ characteristics were recorded: (1) Right or left testis; (2) Presence or absence of acute scrotum as the first symptom. Acute scrotum symptoms included scrotal pain, scrotal swelling, testicular pain, or tenderness [2,5,16-19]. Other symptoms recorded included abdominal pain, vomiting, nausea, diarrhea, and hip pain [16,17,19]. Patients with the first symptom not being acute scrotum were classified into the group “absence” of acute scrotum as the first symptom; (3) The period from first symptom onset to diagnosis (hours). In our institution, all patients were diagnosed with testicular torsion based on sonographic findings. Therefore, the time of the diagnosis of testicular torsion was when an ultrasound examination was started; (4) Testicular salvage or not. Testicular salvage or not was decided based on the surgical findings. In our hospital, manual detorsion was performed during ultrasound examination for all testicular torsion cases. If manual detorsion failed, urgent surgical exploration was performed. In cases where manual detorsion was successful, semi-urgent orchiopexy was performed, resulting in testicular salvage in all successful cases. The decision, testicular salvage or not, was made by pediatric surgeons or urologists with 20, 20, and 25 years of experience in pediatric surgery and urology, and success or failure of manual detorsion was made by these pediatric surgeons, urologists, and radiologists; (5) The period from the diagnosis of testicular torsion to detorsion. The period from diagnosis of testicular torsion to detorsion was recorded. In cases where manual detorsion failed, the time of detorsion was when urgent surgical exploration was performed. In cases where manual detorsion was successful, the time of detorsion was when manual detorsion was performed. Manual detorsion was performed during ultrasound examination; therefore, the diagnosis and manual detorsion were at almost the same time.

Ultrasound examination and sonographic finding

Sonograms were obtained using a linear transducer (9-15-MHz) (LOGIQ 7, E9, E10, and S8; GE Healthcare, Waukesha, WI, USA) by four radiologists with 17, 19, 22, and 29 years of experience, respectively, in pediatric ultrasonography and radiology. All ultrasound examinations were performed within one hour of hospital arrival. The diagnosis of testicular torsion was based on the following findings: the presence of a whirlpool sign at the affected spermatic cord and enlargement with edematous changes in the affected testis [14,15]. Sonographic findings with or without preserved blood flow within the affected testis and heterogenous echogenicity within the affected testis were recorded.

Statistical analysis

Data are presented as mean ± standard deviation. Statistical signiﬁcance was set at P < 0.05 (two-sided). Data were analyzed using a commercially available software program, IBM SPSS Statistics for Windows, version 24 (IBM Corp., Armonk, NY, USA). Patient characteristics and sonographic findings, including right or left testis, presence or absence of acute scrotum as the first symptom, period from first symptom onset to diagnosis and testicular salvage or not, period from diagnosis to detorsion, with or without preserved blood flow within the affected testis, and with or without heterogenous echogenicity within the affected testis, were compared between childhood and adolescence using Fisher’s exact and the Mann-Whitney U tests.

For the testicular salvage rate and first symptom, a single linear regression analysis was used to evaluate the association between each age every one year and both salvage and acute scrotum as first symptom rates. Single linear regression analysis was performed between the time from first symptom onset to diagnosis and patient age.

## Results

Patient characteristics

Patient characteristics are summarized in Table 1, the rate of testicular salvage and first symptom of acute scrotum in each age every one year are summarized in Table 2, and a comparison between the two groups is presented in Table 3.

**Table 1:** Patient characteristics and sonographic findings

Patient characteristics	Values
Total number of patients (n)	58
Patient age (years)	12.13 ± 3.78 (range, 1.2–16.8)
Infant/Adolescent (n%/n%)	11/47
Median (Interquartile Range) age Infant/Adolescent (years)	5.3 (3.31.2–7.4)/13.8 (13.1–14.6)
The period from the onset of the first symptom to diagnosis (hours)	19.97 ± 24.31 (range, 2–72)
First symptom acute scrotum presence/absence (n%/n%)	43/15
Right/Left (n%/n%)	13/45
Preserved blood flow in ultrasound presence/absence (n%/n%)	3/55
Heterogeneous echogenicity within affected testis in ultrasound presence/absence (n%/n%)	11/47
The period from diagnosis to detorsion (hours)	43.45 ± 61.86 (range, 0–180)
Testicular salvage salvage/not salvage (n%/n%)	39/19

**Table 2:** Rate of testicular salvage and first symptom as acute scrotum in each age every one year

Age every one year and total number in this age range	Median age in age every one year (years)	Salvage rate %	Rate of first symptom as acute scrotum %
1–2 years (1)	1.2	0% (0/1)	0% (0/1)
2–3 years (1)	2.1	0% (0/1)	0% (0/1)
3–4 years (2)	3.55	0% (0/2)	0% (0/2)
4–5 years (1)	4.0	0% (0/1)	0% (0/1)
5–6 years (3)	5.4	33.3% (1/3)	66.7% (2/3)
6–7 years (2)	7.6	50% (1/2)	50% (1/2)
7–8 years (0)	-	No patients	No patients
8–9 years (0)	-	No patients	No patients
9–10 years (1)	9.3	0% (0/1)	0% (0/1)
10–11 years (1)	10.6	100% (1/1)	0% (0/1)
11–12 years (4)	11.5	25% (1/4)	50% (2/4)
12–13 years (6)	12.55	83.3% (5/6)	83.3% (5/6)
13–14 years (14)	13.3	71.4% (10/14)	78.6% (11/14)
14–15 years (14)	14.3	92.9% (13/14)	100% (14/14)
15–16 years (7)	15.6	85.7% (6/7)	100% (7/7)
16–17 years (1)	16.7	100% (1/1)	100% (1/1)

**Table 3:** Patient characteristics and imaging findings of testicular torsion between patients in childhood and adolescence

	Infant (number = 11)		Adolescence (number = 47)	P
Time from first symptom onset to diagnosis (hours)	36.18 ± 21.43 (4–72)	vs.	16.17 ± 23.37 (2–72)	0.003
Acute scrotum as the first symptom presence/absence (n%/n%)	3/8	vs.	40/7	<0.001
Right/Left (n%/n%)	0/11	vs.	13/43	0.055
Preserved blood flow in ultrasound presence/absence (n%/n%)	0/11	vs.	3/44	>0.999
Heterogeneous echogenicity within affected testis in ultrasound presence/absence (n%/n%)	(10.91 ± 73.82 (0–180)	vs.	30,00 ± 50.82 (0–150)	0.001
The period from diagnosis to detorsion (hours)	2/9	vs.	37/10	< 0.001
Testicular salvage salvage/not salvage (n%/n%)	2/9	vs.	37/10	<0.001

Overall, 58 patients were included in the study. Of these, 47 and 11 lesions were located on the left and right sides, respectively. Testicular torsion occurred in 11 and 47 patients in childhood and adolescence, respectively. The average time from first symptom onset to diagnosis was 19.97 ± 24.31 (range, 2-72) hours, while that from diagnosis to detorsion was 43.45 ± 61.86 (range, 0-180) hours. The presence of acute scrotum as the first symptom was observed in 43 of the 58 patients. Only three patients had preserved blood flow within the affected testis on ultrasound, 11 had heterogenous echogenicity within the affected testis, and testicular salvage was accomplished in 39/58 patients.

Comparison of patients’ characteristics between childhood and adolescence

Significant differences were observed in time from first symptom onset to diagnosis (hours) between childhood and adolescence (36.18 ± 21.43 (4-72) vs. 16.17 ± 23.37 (2-72) hours, respectively [P = 0.003]); in the period from diagnosis to detorsion (10.91 ± 73.82 (0-180) vs. 30.00 ± 50.82 (0-150) hours, respectively [P = 0.001]); presence/absence of acute scrotum as the first symptom (3/8 vs. 40/7, respectively [P < 0.001]); presence/absence of heterogeneous echogenicity within the affected testis (5/6 vs. 6/41, respectively [P = 0.025]); and testicular salvage/not salvage (2/9 vs. 37/10, respectively [P < 0.001]). No significant differences were observed between the childhood and adolescence groups in lesion location (right/left: 0/11 vs. 13/43, respectively [P= 0.055]) and presence/absence of preserved blood flow on ultrasound (0/11 vs. 3/44, respectively [P > 0.999]).

Association between patients’ characteristics and age

**Figure 1:**
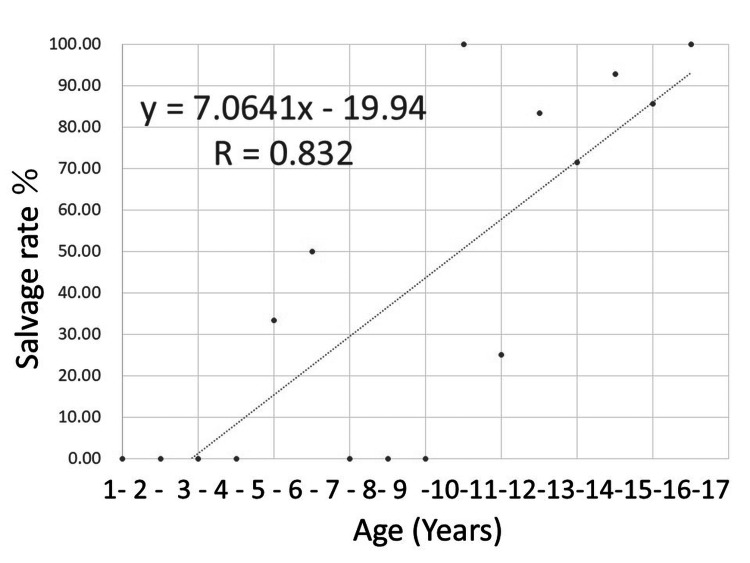
Scatter plot of testicular salvage rate in each year every one year Based on the single linear regression analysis, a strong positive correlation was observed between the testicular salvage rate and patient age every one year (r = 0.832, P < 0.001).

**Figure 2:**
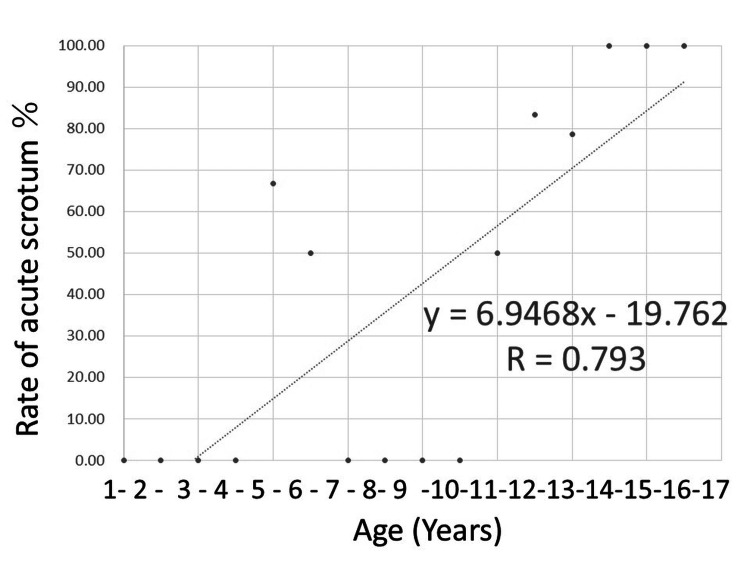
Scatter plot of rate of first symptom as acute scrotum in each year every one year Based on the single linear regression analysis, a strong positive correlation was observed between acute scrotum as the first symptom rate and patient age every one year (r = 0.793, P = 0.001).

**Figure 3:**
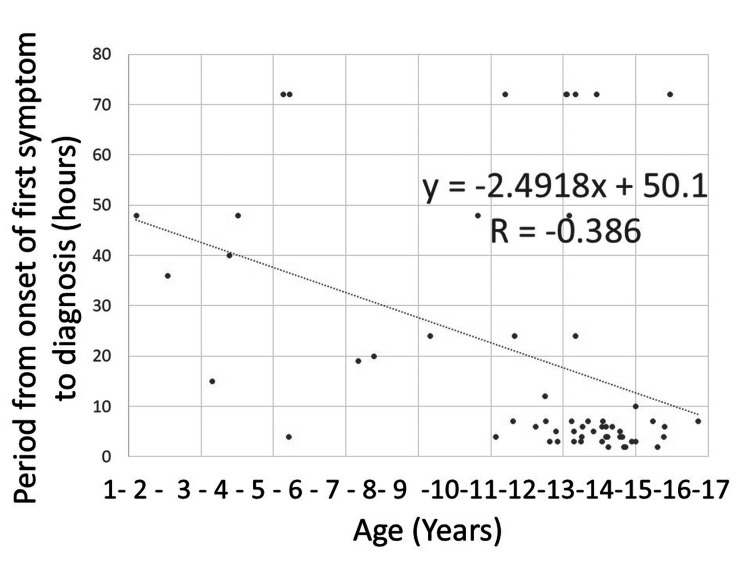
Scatter plot of rate of period from onset of first symptom to diagnosis Based on the single linear regression analysis, a weak negative correlation was observed between time from first symptom onset to diagnosis and patient age (r = -0.386, P = 0.003).

Figures 1, 2 show the results of a single linear regression analysis plotting the testicular salvage and acute scrotum as first symptom rates in relation to each age every one year. Figure 3 shows the results of a single linear analysis between the time from first symptom onset to diagnosis and patient age. Strong positive correlations were observed between patient age every one year and both testicular salvage and acute scrotum as first symptom rates (r = 0.832, P < 0.001; and r = 0.793, P = 0.001; respectively), while weak negative correlations were found between time from first symptom onset to diagnosis and patient age (r = -0.386, P = 0.003). The “r” indicates +1.0 to 0.8 or -1.0 to 0.8, which means perfect or very strong association, +0.8 to 0.6 or -0.8 to -0.6, which implies strong association, +0.6 to 0.4 or -0.6 to -0.4, which signifies moderate association, +0.4 to 0.2 or -0.4 to -0.2, which represents weak association, and +0.2 to 0.0 or -0.2 to 0.0, which shows very weak or no association, respectively.

## Discussion

Testicular torsion in childhood tends to present without typical symptoms, such as acute scrotum, leading to a delay in diagnosis of testicular torsion, heterogeneous echogenicity within the affected testis, and low salvage rates for the affected testis. These results indicate that even if pediatric patients, especially in childhood, have abdominal symptoms or hip pain, which are the most frequent complaints in the pediatric emergency department [1,2,18], physicians should consider the possibility of testicular torsion. A physical examination of the scrotum might be useful to avoid overlooking testicular torsion during childhood.

A previous study showed that prepubertal patients (aged 1-12 years) had a greater tendency to have abdominal symptoms, not an acute scrotum, than post-pubertal patients (aged 13-16 years) [8]. Additionally, younger patients were less likely to exhibit typical symptoms of acute scrotum than older patients. The following three nerve groups influence the spermatic cord plexus: the rostral (derived from the aortic and renal plexuses to the spermatic cord and testis), the intermediate (from the proximal portion of the superior hypogastric plexus to the spermatic cord, epididymis, and proximal vas deferens), and the caudal (from the distal ureter to the vas deferens, epididymis, and seminal vesicles) [17,20]. Consequently, testicular pain should be differentiated from abdominal, kidney, and ureteral pain. However, in childhood, the testis, spermatic cord, epididymis, seminal vesicle, and vas deferens may be less mature than in adolescence, making it challenging to distinguish testicular pain from abdominal pain. Therefore, atypical symptoms, including nausea, vomiting, and abdominal pain, may present in infants with acute scrotum [16-19].

Heterogeneous echogenicity within the affected testis has been reported to be a useful indicator for testicular loss. Therefore, this finding was more commonly detected in childhood than in adolescence. Additionally, all cases of testicular loss had surgical exploration after the failure of manual detorsion; therefore, the period between the diagnosis and detorsion was longer than that in cases with successful manual detorsion.

In our cohort, only three patients in adolescence had preserved blood flow within the affected testis, and their testes were salvaged. This finding was not observed in patients in childhood. Testicular size is usually smaller in childhood than in adolescence; therefore, visualizing weak blood flow within twisted testes may be challenging [21,22]. Nonetheless, this finding suggests that the testis is not completely necrotic and could still be salvaged; therefore, urgent interventions are crucial to prevent testis necrosis.

Despite demonstrating the importance of atypical symptoms in infants with testicular torsion, this study had some limitations. First, this was a retrospective study with a small sample size, particularly the patients in childhood. Second, we did not evaluate testicular function after salvage. Testicular atrophy has been reported following surgical detorsion [23]. Given the importance of testicular function in pediatric patients, long-term follow-up is necessary to assess this function. Further studies involving a large number of patients with extended follow-up periods are required to evaluate testicular function. Third, ultrasound quality depended on the operators’ skills. In pediatric patients, especially in childhood, the small size of the testis makes evaluating its vascularity technically difficult. In our study, all ultrasound examinations were performed by well-experienced pediatric radiologists; however, in children with clinically suspected testicular torsion without abnormal sonographic findings, it was difficult to diagnose testicular torsion. Other modalities, including magnetic resonance imaging, may be useful in diagnosing testicular torsion [24].

## Conclusions

Testicular torsion in childhood tends to present with atypical symptoms such as abdominal pain or hip pain, not acute scrotum. It may result in diagnostic delay and low salvage rates for the affected testis. Physicians should consider the possibility of testicular torsion when examining children with abdominal pain, and additional physical examination of the scrotum might be useful to detect testicular torsion in the early phase.
